# Educating patients in a French cancer treatment center: How to ensure therapy safety while reckoning patients’ knowledge and power to act

**DOI:** 10.1371/journal.pone.0304899

**Published:** 2024-06-06

**Authors:** Charlotte Bruneau, Jean-Paul Genolini, Philippe Terral

**Affiliations:** Center for Research in Social Sciences, Sports and Body, University Paul Sabatier Toulouse, Toulouse, France; Pan African University of Life and Earth Sciences Institute, PAULESI, NIGERIA

## Abstract

In this article, we analyse how health professionals educate cancer patients to care for their condition and keep strict control over therapy safety. We study how much room for negotiation is left to patients during medical consultations so resources can still be exchanged. We pay particular attention to the trade of knowledge and powers between patients and doctors (power to act and to express oneself in an imbalanced relationship where knowledge is unequally shared). We opted for a qualitative approach with 41 interviews and several ethnological observations, first of consultations in haematology, then of pre-planned phone calls made to patients during the course of a cancer therapy follow-up scheme. The declared ambition of turning cancer patients into self-responsible patients actually re-enacts well-known procedures of control and knowledge acquisition aimed at narrowing their margin of manoeuvre for the sake of therapy safety. Even if some freedom is conceded, patients remain under the control of their medical hierarchy. Health professionals privilege two methods to keep control over patients and teach them therapy safety procedures. Which method is chosen, and how it is used, is dictated by the relationship between socially-diverse patients and health professionals. In the end, what the patient learns and the amount of control the doctor keeps over this process will depend on the distribution of power and knowledge among them, but asymmetry will always remain.

## Introduction

The field of health has lately paid a lot of attention to patients’ individual experience, merely as a response to the emergence of the “21^st^ century patient” and of its offshoots, the expert patient, the layman patient and the patient partner [1–6]. As a consequence, at every level of the health system, encouraging patients’ involvement has been considered key to improve global health and the quality of care [7]. It is in this context that the concept of “health democracy” settled down, namely in France where it was buoyed by a string of laws and charters (Ottawa Charter of 1986, Kouchner law of March, 4th, of 2002, HPST law of July, 21th, of 2009) aimed at making patients more autonomous and self-responsible. This apparent redistribution of medical knowledge and power went along a wave of new treatments such as OAA or Oral Anticancer Agents, a type of chemotherapy that can be administered to patients directly at their home, which avoids long hospital stays. Such innovation has affected cancer therapy both in time and space, turning it into an outpatient (or ambulatory) therapy. Education to health becomes then even more crucial as cancer patients find themselves “responsible” for the timely and safe administration of their OAA [8], as well as for the efficiency of the therapy. Consequently, as the geographical distance between patients and health facilities only increases, health professionals cannot fully guarantee therapy safety anymore. They are not in a position to prevent medical hazards caused directly or indirectly by the therapy, since these events occur in patients’ homes [3]. So as relations between doctors and patients are reshaped and their respective roles reshuffled, new working tools are conceived, first to improve how secondary effects are dealt with, then to ensure that patients stick to their treatment and, broadly-speaking, take heed of their new status [9–11]. Among these tools, we find a range of follow-up schemes directed at patients undergoing ambulatory and oral treatments. Thus our research questions how knowledge (both patients’ and health professionals’) and power (power to act and to express oneself, inequality of power between individuals and their respective knowledge) are traded during medical consultations.

We established that there are two main ways for health professionals to teach cancer patients to care for their disease in order to ensure therapy safety. The first way is based on the minimal level of mutual understanding that exists between patients and doctor [12]. This method is nothing more than a re-enactment of the “operatory chain-processes” found in medical consultations, which for their part are familiar to all. Resting on the asymmetry of knowledge between patients and health professionals, these “operatory chain-processes” are an adaptation of the medical consultation’s original script (“introduction, health check, results, discussion, report, prescription, closure”, [13]) and leave patients with almost no room to negotiate or power to act. The second way to teach patients relies on their ability to deal with these “chain-processes” in which the doctor is a “figure of authority” [14]. Once a certain level of familiarity has been reached, a compromise becomes possible between both parties. In this case, patients have the power to act for their own health. How well do patients and doctor adjust to each other than depends on their willingness to find a common ground with the ultimate aim to ensure therapy safety. Once this condition is fulfilled, an agreement on the matter can be sealed [15]. We paid particular attention patients’ power to act, i.e. to the room left for negotiation in medical consultations and to this process of mutual adjustment, both of which allow patients and doctors to trade knowledge and power. So the path chosen to ensure safety therapy will depend on how the relationship unfolds during consultations and how knowledge and power is distributed. Indeed, consultations are usually asymmetric in order for doctors to ensure a safe access to knowledge for their patients.

Thus on the one hand, we will describe the way in which giving patients responsibility over their condition eventually perpetuates the control applied over them by doctors, whose superiority is acknowledged by both parties. As long as therapy safety is at stake, patients willingly allow doctors to shrink the room available to negotiation. On the other hand, the introduction of new learning schemes present patients with fresh opportunities for power to act, including negotiation within the “chain-processes” [13] of what is socially defined as a “medical consultation”. Nevertheless, such freedom is conceded only provided it remains under medical control.

## Materials and methods

This study was part of our PhD thesis (CB) about the acquisition of expertise by the patients of a French cancer treatment centre. Our interest was how medical consultations help patients gather the knowledge necessary to care for their cancer in a way that is cognisant of therapy safety control. Our data is the result of two years of investigation by PhD student in sociology, experienced in qualitative methods (CB). It comprising interviews and ethnological observations, first of medical consultations, then of phone calls made to patients as part of a therapy follow-up scheme. The follow-up scheme is presented in Table 1. The qualitative approach suited our issue well since it focuses on individual experience, on interpersonal relationships and, more widely, on everything that cannot be quantified nor measured [16].

However, opting for a qualitative approach in the field of health comes with ethical stakes. Ethical approval was granted by the French National Data Protection Authority (Ref. N°918239) and the University of Toulouse Research Ethics Committee (Ref. N°2018–092). Participants were recruited by a haematologist during the investigating researcher’s observation time (CB). The doctor presented the study to the patients and distributed an informed and consent form. All participants signed an informed consent form before data collection. Recruitment period started on April 6, 2019 and ended on June 15, 2020.

**Table 1:** Characteristics of the cancer therapy follow-up scheme.

This scheme is performed by a French cancer treatment centre. Follow-up is done through phone calls and comes in the wake of medical consultations in haematology. Patients are introduced into this scheme at their haematologist’s request, and see it simply as a continuation of their therapy. Follow-up phone calls were made to patients from the cancer treatment centre where we led our study, and where a dedicated call centre was created in 2006. The scheme ran on public funds (Agence Nationale de la Recherche, aka ANR) in its first five years before the cancer treatment centre took over financial responsibility. Patients included in the scheme belong to two categories: either they start an intravenous cancer therapy, or they are administered OAA after their cancer has relapsed. Patients receive systematic and pre-planned calls during the active phase of their treatment. The stake here is to maintain the overall quality of treatment even from a distance, which requires from patients and visiting health professionals (visiting nurses or family doctor) to acquire a specific set of skills. More precisely, follow-up calls are destined to make sure that home-bound patients take their highly toxic cancer therapy safely and timely. Besides that, patients under OAA are enrolled in a specific research on the toxicity of two targeted therapies against malignant blood tumours (ibrutinib and idelalisib). Two coordinating nurses were working full time for the follow-up scheme which included 90 to 100 patients day in day out. Phone call frequency varied according to treatment. Patients under intravenous chemotherapy were called twice a week for six months. Intravenous chemotherapy courses, spaced fifteen days to three weeks apart, are administered on an outpatient (or ambulatory) hospitalization for four to six months. The frequency of calls means that nurses are able to follow patients during the periods between courses, when aplasia and adverse effect (tiredness, hair loss, etc.) occur at home. Patients under OAA were for their part called every two weeks for six months, and then once a month during the next semester. OAA can be prescribed after a first line of intravenous chemotherapy. As a result, patients already have some experience of cancer treatment, enabling nurses to reduce the frequency of calls. After one year of oral treatment, patients are judged competent enough to manage the potential side effects of OAA at home (heart rhythm disorder, diarrhea, arthralgia, hematomas, etc.). Patients were also required to take a blood test on the day before they received a call. Pre-planned (or in-protocol) calls were made on Wednesday and Friday mornings. The rest of the week was devoted either to attend patients’ calls (“non-planned” or “out-of-protocol” calls); to in-protocol calls that were not answered or postponed; to complete the forms that nurses must fill during each conversation with a patient. These forms contain relevant information such as identity (of the patient, of its relatives, of its family doctor), treatment type, cycle number, secondary effects and so on. Once filled, they are sent to the patients’ haematologist. Most of in-protocol calls start with general questions about mental and physical health, family and daily life (work, leisure), after which nurses check patients’ weight and blood test results, and ask if there is any secondary effect. If it’s the case, they transmit the doctor’s recommendations to keep secondary effects under control (extra exams or treatments or even a pause in the treatment might be required). The call closes on a recap of the next exams (blood test, medical consultation, radiology). These calls help to enhance OAA adherence, improve the transitional efficiency of information, mitigate the patient’s social isolation at home and prevent patient re-hospitalization through direct management of side effects, thus avoiding the harmful progression of toxicities to ensure therapy safety.

### Direct observations

Our study material comprises a whole year of ethnographical non-participatory observations of haematology consultations in a cancer treatment centre (310). The investigating researcher (CB) was presented as a sociology student working on cancer patients experience. No relationship was established with participants before the start of the study. To push our study further, we also observed the follow-up calls made to these patients (178). Focusing on patients was key to better understand therapy safety control procedures and the skills they require from them and/or develop in them. Furthermore, the ethnographical approach provides a necessary counterpoint to interviews. It allows us to confront individual testimonies with priceless soundbites and real-life situations revealing how cancer patients acquire knowledge.

### Interviews

To complement our observations, we interviewed 24 patients suffering from malignant blood tumours and 12 health professionals (three nurses, two haematologists, two clinical research assistants, one oncologist, one family doctor, one hospital administrator and two pharmacists, one from a hospital, the other from a local pharmacy). They were recruited at the cancer treatment centre and patients during haematology consultations in face-to-face. These interviews provided better understanding of how patients and health professionals work together and trade knowledge over the course of the follow-up scheme. All in all, we led 41 interviews which lasted 1 hour and 32 minutes on average. In order to understand how therapy safety control is managed, we focused on the following topics: patients’ education to cancer care; patients’ relationship with health professionals; patients’ approach of medical knowledge. Twenty-four patients took part. They were 13 women and 11 men, aged between 49 and 76 (67 on average), majority from the middle class and the upper tiers of society, detailed in Table 2. The selected socio-professional categories follow the INSEE (French National Institute for Statistics) nomenclature issued in 2003. More than half of the patients interviewed were retired. In their case, we took into account the most recent occupation.

**Table 2:** Classification of patients interviewed according to their gender and socio-professional category.

	Male (11)	Female (13)	Overall (24)
Upper class (categories 3 and 4)	8	7	15
Middle class (categories 1, 2 and 5)	2	4	6
Lower class (categories 6 and 8)	1	2	3

### Data collection

Our study material comprises a whole year of ethnographical observations of haematology consultations in a cancer treatment centre (310). To push our study further, we also observed the follow-up calls made to these patients (178). The researcher’s prolonged commitment in the field and persistent observations are particularly important in producing the rich descriptions and thick data, needed to ensure the validity of our qualitative methodology [17]. Focusing on patients was key to better understand therapy safety control procedures and the skills they require from them and/or develop in them. Furthermore, the ethnographical approach provides a necessary counterpoint to interviews. It allows us to confront individual testimonies with priceless soundbites and real-life situations revealing how cancer patients acquire knowledge.

To complement our observations, we interviewed 24 patients suffering from malignant blood tumours and 12 health professionals (three nurses, two haematologists, two clinical research assistants, one oncologist, one family doctor, one hospital administrator and two pharmacists, one from a hospital, the other from a local pharmacy). Interview guide were designed to provide better understanding of how patients and health professionals work together and trade knowledge over the course of the follow-up scheme. The questions are given in Table 3. In order to understand how therapy safety control is managed, we focused on the following topics: patients’ education to cancer care; patients’ relationship with health professionals; patients’ approach of medical knowledge. Patients’ recruitment was continued until data saturation was reached, i.e. until the new data was redundant, no longer teach the investigator anything and contradict his analytical framework [18, 19].

**Table 3:** Participant interviews’ topic list.

Topic list	Sample questions
Biographical information	Can you introduce yourself?
Onset of illness	Can you tell me about the period when your illness began? How did you experience the diagnosis? How was your course of cancer care? How did you handle recurrence (if applicable)?
Provider-patient relationship	How are your relationships with healthcare providers? Did you feel involved in the treatment choices? Do doctors consider your opinion into account? Have you faced difficulties understanding doctors’ explanations?
Organizational skills	How do-you manage the administrative aspect of your cancer care? How are you helped in your daily life?
Follow-up phone calls	How did you hear about the follow-up phone calls? What do you think of this follow-up scheme? What have you learnt from this follow-up scheme?
Patient education	Do you feel that you receive sufficient explanations about your illness and treatments? Do you educate yourself about your illness? In what ways? What have you learned during your cancer care? Would you like to learn more?

### Data analysis

Throughout the investigation, we wrote down in a 161-page-long computer diary everything we observed, from medical consultations to follow-up phone calls. Interviews were recorded in agreement with the interviewees and the full verbatim was transcribed on computer “so as to respect the speech in shape and form” [20]. The student PhD (CB) did a manual thematic analysis of interviews and observations [21]. Data was broken down into extracts, which be categorized into 17 categories in order to identify elements relating to patients’ education (social characteristics, disease and treatment, relationship to the disease, to the other health professionals, to knowledge, etc.). This category’s coding system was shared with the other co-investigators to improve the validity and certainty of the results. We focused on examining thematic categories influence based on different patients or situations. Special attention was given to compare reccurrences and negative cases to ensure analysis rigor and reliability.

## Results and discussion

### Characteristics of participants

Tables 4 and 5 presents the participants demographics. Two overarching themes were identified, relating to the mechanisms through health professionals to teach cancer patients co care their disease in order to ensure therapy safety: 1) asymmetry key for doctors to keep control, and 2) devolution of power yes, but under tight control.

**Table 4:** Characteristics of the patients’ studies (n = 24).

Name	Sex	Age	Living space	Family situation	Level of education	Job	Cancer care
Patrick	Men	61	Half-Urban	Divorced	NVQ/BTEC First Diploma/High school	In disability Sales technician	Diagnosed in 2009 2 recurrences in 2013 and 2018 In treatment at time of interview with OAA
Jacques	Women	76	Rural	Divorced, children	Higher British A-level	Retired PE teacher currently Mayor (3terms)	Diagnosed in 2008 2 recurrences, including one in 2016 In treatment at time of interview with OAA
Christiane	Women	71	Rural	Divorced, 2 children	Higher British A-level	Retired Journalist	Diagnosed in 2001 2 recurrences in 2002 and 2014 In treatment at time of interview with OAA
Bernard	Men	75	Rural	Divorced	NVQ/BTEC First Diploma/High school	Retired Cook	Diagnosed in 1995 In treatment at time of interview with OAA
Sylvie	Women	58	Urban	Maried, 3 children	Higher British A-level	Active Civil servant Full time Documentalist	Pas de date renseignée pour le diagnostic 1 recurrence in 2016 In treatment at time of interview with OAA
Martine	Women	70	Urban	Maried, children	Middle school	Retired No professional status Never worked	Diagnosed in 2014 1 recurrence in 2017 In treatment at time of interview with OAA
Daniel	Men	66	Urban	Maried, 2 children	NVQ/BTEC First Diploma/High school	Retired Electrician EDF, supervisor and technician in the movement of energy and transport of electricity	Diagnosed in 2006 1 recurrence in 2017 In treatment at time of interview with OAA
Françoise	Women	70	Urban	Divorced, 1 child	British A-level	Retired Banker	Diagnosed in 2007 2 recurrences in 2014 and 2017 In treatment at time of interview with OAA
Annick	Women	73	Urban	Maried, 2 children	Middle school	Retired Civil servant in post office	Diagnosis date unknown 1 recurrence in 2016 In treatment at time of interview with OAA
Brigitte	Women	60	Urban	Maried, 3 children	Higher British A-level	Retired School teacher	Diagnosed in 2013 In treatment at time of interview with OAA
Alain	Men	69	Half-Urban	Divorced, 1 child	British A-level	Retired Insurance agent	Diagnosed in 2013 1 recurrence in 2015 In treatment at time of interview with OAA
Philippe	Men	54	Half-Urban	Maried, 2 children	Higher British A-level	Working Permanent contract Full time Radio manipulator	Diagnosed in 2008 2 recurrences in 2016 and 2018 In treatment at time of interview with OAA
Jean	Men	72	Urban	Maried, children	NVQ/BTEC First Diploma/High school	Retired Construction supervisor	Diagnosed in 2011 1 recurrence in 2018 In treatment at time of interview with OAA
Hélène	Women	60	Urban	Divorced, 1 child	Higher British A-level	Job seeker International shipping, export assistant, advertising agency, desktop publishing, day job	Diagnosed in 2013 In treatment at time of interview with OAA
Valérie	Women	50	Urban	Maried, 2 children	Higher British A-level	Active Civil servant Full time Post office counter, currently in debt recovery agency	Diagnosed in 2005 Fifteen or so recurrences In treatment at time of interview with OAA
Corinne	Women	59	Urban	Maried, 2 children	Higher British A-level	Active Civil servant Full time Accountant, reconversion in tourism, in teacher, currently educational consultant	Diagnosed in 2016 In treatment at time of interview with OAA
Nathalie	Women	49	Rural	Maried, 3 children	Higher British A-level	Working Permanent contract Full time Nursing assistant	Diagnosed in 2012 Under medical surveillance, never treated at time of interview
Charles	Men	67	Unknow	Maried, 3 children	Higher British A-level	Retired IT technician	Diagnosed in 2008 1 recurrence in 2016 In treatment at time of interview with OAA
Frédéric	Men	67	Unknow	Unknow	Higher British A-level	Working Permanent contract Full time Asset manager	Diagnosed in 2008 2 recurrences between 2010 and 2019 Under medical surveillance at time of interview
Luce	Women	64	Urban	Children	Higher British A-level	Retired SEN teacher	Diagnosed in 2012 Under medical surveillance at time of interview
Gérard	Men	Unknow	Urban	Unknow	NVQ/BTEC First Diploma/High school	Retired Technical inspector and building health and safety coordinator working	Diagnosed in 1998 3 recurrences between 2004 and 2015 In treatment at time of interview with OAA
Jeanne	Women	Unknow	Urban	Maried, children	Higher British A-level	Working Craftsman Full time Florist	Diagnosed in 2009 In remission for 2 years at time of the interview
Mireille	Women	55	Urban	Divorced, children	NVQ/BTEC First Diploma/High school	On a training course Court-appointed receiver	Diagnosed in 2009 In remission for 2 years at time of the interview
Pierre	Men	68	Unknow	Maried, 2 children	Higher British A-level	Retired Public finances	Diagnosed in 2011 2 recurrences in 2013 and 2015 In treatment at time of interview with OAA

**Table 5:** Characteristics of the professionals’ studies (n = 12).

Name	Sex	Level of education	Diplome(s)	Job
Catherine	Women	Higher British A-level	Bachelor of Nursing, BTEC Higher Nation Diploma in Therapeutic Patient Education, Master’s Degree in Care Coordination	Care Coordination Nurse
Marie	Women	Higher British A-level	Bachelor of Nursing	Care Coordination Nurse
Julie	Women	Higher British A-level	Bachelor of Nursing, BTEC Higher Nation Diploma in Palliative care and Skin toxicity, Bachelor’s Degree in Public Health, 40 hours of training in Therapeutic Patient Education	Hospital Nurse
Michel	Men	Higher British A-level	PhD in Medicine	MD-PhD in Haematology
Arnaud		Higher British A-level	PhD in Medicine	MD-PhD in Haematology
Marianne	Women	Higher British A-level	PhD in Medicine, 40 hours of training in Therapeutic Patient Education	MD in Oncology
Louis	Men	Higher British A-level	PhD in Medicine, 40 hours of training in Therapeutic Patient Education	MD
Suzanne	Women	Higher British A-level	Bachelor of Nursing, BTEC Higher Nation Diploma in Therapeutic Patient Education	Hospital administrator
Mélanie	Women	Higher British A-level	Master in toxicology, BTEC Higher Nation Diploma in clinical research	Clinical research assistant
Caroline	Women	Higher British A-level	PhD in Sciences of pharmacological innovation	Clinical research assistant
Isabelle	Women	Higher British A-level	PhD in Pharmacy, 40 hours of training in Therapeutic Patient Education	Hospital Pharmacist
Léa	Women	Higher British A-level	PhD in Pharmacy, 40 hours of training in Therapeutic Patient Education	Community pharmacist

### Asymmetry key for doctors to keep control

Home-administered cancer therapy keeps patients away from health facilities and in-person appointments. Medical consultations in haematology, which take place every three or four months in average, have therefore become the only moment when doctors and homebound patients are face to face. However, even if patients are more self-responsible than before, they are left with a slim margin of manoeuvre, precisely in order to ensure the safety of their therapy. Health professionals use learning methods to equip patients with medical to pass on medical guidelines of good practice to patient, thus equipping them with the behaviours and skills required to account for their own safety. These methods can be described as “traditional” ones. Doctors activate socio-cognitive processes that derive from traditional methods of control based on the asymmetric relationship between patients and their doctor, whose domination is a prerequisite to mutual trust. We saw doctors taking great pains to translate into laymen’s terms medical notions that are key to disease oversight. Doctors take as a principle that ideas worded simply are easily remembered, whereas long explanations only lead to confusion, and technical terms to fear [22]. Therefore, doctors express their ideas in a “simple lexicon” and illustrate them with metaphors that are accessible to everybody. One example for this is “Lymphocytize yourself”, an educative printed cartoon about blood cancers distributed to patients when they enter therapy.

“Doctors try to give explanations to their patients. Usually, they succeed by using simple words. But they won’t explain how ibrutinib (a type of OAA used against some leukaemia) works, nor say that “it’s a protein kinase inhibitor”. Instead they draw sketches, which I think is cool. So most patients understand lots of things, especially in blood biochemistry. Most of them come up to us and say: “my blood parameters have improved”, because they know what to read.”

(Mélanie clinical research assistant)

Once familiarized with blood biochemistry, patients are able to detect abnormalities in their blood and thus self-monitor their health. There are critical thresholds in the blood that indicate if a disease recedes, spreads or stagnates, so it’s crucial that patients can identify them. They are thus encouraged to “signal any loss of control or feeling of powerlessness to keep any danger or risk in check” [23]. Trained to become their personal whistle blowers and as such, the first link in a therapy safety chain, patients soon realize that responsibilities are neatly divided. The doctor is in charge of the disease while they are in charge of their daily life, hence of the safety of their therapy. Nonetheless, according to some patients, being able to detect “that something is wrong” in their blood biochemistry and that “something needs to be done” doesn’t mean that they understand the “process” that lies behind.

“I don’t understand anything. I just notice when something is wrong because there is an asterisk or a dot, but I don’t know what it means. This is also why I put all my trust in the doctor because I hope that if something goes awry, he will be able to see it. That he won’t miss it. Do you really think that I know what “creatine level” means? [noise expressing ignorance] Of course I don’t know. Of course not.”

(Sylvie cancer patient)

Patients don’t need medical knowledge to avoid harming themselves when they are not under direct medical oversight, they just need to know the norms. For everything else, they rely on a network of experts who give meaning to the data. It’s as if doctors assumed ownership of the sickness whereas patients resume their daily life. This “traditional” learning method is actually a re-enactment of well-known forms of control in which green and red lines are neatly drawn, and patients’ power to act is restricted.

To complement this “traditional” education, doctors enrol patients in the follow-up scheme and we noticed that to convince them, they activate the classical script of a medical consultation. In other words, they do everything to make sure that the relationship between them and patients remains asymmetric, which is seen as key to ensure therapy safety. Concretely, during the time of our study, a clinical trial was underway to assess the toxicity of two targeted therapies (namely ibrutinib and idelalisib) against blood tumours. Haematologists presented that trial to their patients as complementary to consultations and praised it as “a tailor-made follow-up” or a “tight network” thanks to which “patients are closely followed while safely staying at home”. According to the health worker who implemented the scheme at the cancer treatment centre, “the main asset of our scheme is that each patient is individually taken care of. It gives patients a huge sense of security.” This sense of security is provided by four layers of medical procedures all aimed at monitoring patients: the follow-up scheme itself; an increase in the number haematology consultations; more frequent medical exams (blood tests, MRI or PET-scans); and life quality questionnaires. According to doctors, this intricate web of medical procedures is a guarantee of safety and for this reason, they actively encourage patients to enrol in the follow-up scheme. Patients often even give written consent without being fully informed.

“When the doctor told me that I had to take that therapy [ibrutinib], […] he also gave me these documents. He gave me… this [she shows us the instruction sheet for the follow-up scheme, which also serves as written consent for enrolment]. It was about imbruvica. And then he said “please sign down, you’ll read the contents later, on the train back home”. And then I did realize that these documents were the actual written consent form. And he as saying “please sign down”. You know… he was on a rush, as always, so he gave me all that stuff and said “you’ll read that on the train back home”, “you’ll read that later”. […] I mean, OK, [doctors] don’t have a lot of time. They already are under a great load. And in that thing [the form], everything is of course said with all the right words and niceties, you’re told that you can agree to this, to that… yet it was a bit too… sped up. But once in the train, I could not go back to the hospital. […] Even if it was only there that I worked out what it was all about. I said “oh gosh”. I mean, it was way too late to argue. But [ibrutinib] worked so fantastically on me that I don’t nurse any grievance against him anymore..”

(Hélène cancer patient)

As Hélène testifies, patients and doctors adjust their respective positions to facilitate enrolment, sometimes unconsciously. And even when they are aware of being manipulated by the doctor, patients still give in to the whole “follow-up machine” because they see in the follow-up scheme a logical continuation of medical consultations. This is why they prefer to agree to something they don’t know the contents of in order not to delay their enrolment, instead of reneging and starting negotiations with the doctor. Those adjustments, as we said earlier, are mostly unconscious. To patients, all the processes associated with cancer therapy seem to flow naturally or logically [24]. This apparent fluidity favours and even reinforces the asymmetric relation between patients and health professionals.

Henceforth the use of well-known medical scripts that reproduce the traditional medical hierarchy and put doctors in the driver’s seat. It’s from this premise that trust is built between them and patients. The follow-up scheme in turn reinforces this trust, emphasizing and ensuring therapy safety at the expense of the patients’ margin of manoeuvre.

### Devolution of power yes, but under tight control

Even if it is drastically reduced by the learning methods used to ensure therapy safety, some room remains for patients to negotiate, albeit within the boundaries of the socially-accepted asymmetric medical consultation in which patients’ access to knowledge is controlled. However, it sometimes emerged during consultations that patients and doctors can reach a level of mutual understanding for patients’ voices to be heard. Those suffering from a post-chemotherapy relapse can for instance give their opinion about the next treatment, even if the options presented were previously chosen by the doctor. Indeed, we heard doctors telling their patients that OAA can be administered only after a first round of chemo, otherwise they lose efficiency on the long-term. So there can be negotiations but once scientific evidence is brought to the table, it kills all debate. As one clinical research assistant puts it, “yes, patients can choose. More or less. Their options are restricted because there are instructions to follow, after all.” What appears to be a slight concession to patients under the guise of freedom of choice happens to remind them of their responsibility, which is to stick to their treatment according to the rules of therapy safety. At no moment doctors relinquish their control and most of the times, patients don’t even know what they’re being invited to negotiate.

But we found that a majority of patients still won’t exploit the limited amount of freedom they are allotted. It’s especially the case for lower-class patients, who find comfort in an asymmetric relationship with the medical world since they just have to hand themselves over to doctors. According to them, “the doctor is on one side of the fence, the patient on the other. There is no crossing that fence. Patients must stick to their role and not even question therapeutic choices.” Patients from middle and upper classes also feature in that group, but their position is slightly different. If they don’t engage into even limited negotiations, it’s because their “chronic illness trajectory” [25] can be described as “simple”. Their therapy is “comfortable” since it doesn’t prevent them from leading a “normal life”.

“[My treatment] is not a heavy one, a single pill every morning. So yes, I have a fairly normal life. Taking my pill has become like a habit or a reflex. I don’t even notice it anymore. […] It is so much more comfortable than for example an organ transplant.”

(Patrick cancer patient)

Why do these patients from all walks of life deny themselves the opportunity to have a say about their therapy? Mainly because it goes against what they expect of a medical consultation during which “we’re not asked anything. On the contrary, everything is just imposed on us.” It is less costly for patients to rely on medical expertise, hence to let doctors decide for everything health-related, than to try to have their say in how their disease is being treated.

“When I’m under [the doctors’] care, I do what they tell me. When my cancer relapsed, Arnaud [the doctor] asked me to choose between chemotherapy and an oral therapy. But I don’t have the relevant information… I mean, I’m not competent to make such a decision. […] So he let me choose my treatment but eventually I did what he wanted me to do. […] It’s like when you take your car to the mechanic. The mechanic is the one who knows what to do and who are we to contradict? We don’t have the necessary skills. […] To me it feels a bit like the doctor holds all the knowledge, like teachers when we were kids. Doctors hold the knowledge and patients are neophytes. Not neophytes, philistines even. At least this is how I see myself as a patient. Patients and doctors belong to two different worlds entirely.”

(Sylvie cancer patient)

This testimony from an upper-class woman is symptomatic of a medical hierarchy that feeds on asymmetry of knowledge and power imbalance to perpetuate itself. Doctors exert a rational authority [26] that’s based on status, experience and skill, and gets legitimized by the institution they belong to [27]. Health professionals concede that patients “follow doctors’ advice just because they trust their doctor! A doctor is a doctor!” This “trust” stems from a situation in which doctors are “experts” who take charge of everything, and patients “ignoramus” who don’t ask questions. All of this amounts to a situation in which doctors are “authority figures” [14] who know all that needs to be known about cancer therapy [28], and where patients’ participation is considered too time-consuming to be bothered with. As a result, everything falls along familiar lines, with patients taking care of their daily lives and doctors taking care of the illness, eventually restricting patients’ margin of manoeuvre.

The follow-up scheme, initially designed to give more leeway to the patient, eventually reproduces more often than not the same asymmetry than medical consultations. We indeed found by observing several phone calls between patients and nurses that the former are presented with two overlapping learning methods that, once combined, control their access to knowledge. On the one hand, thanks to phone conversations and the delivery upon enrolment of a handbook called “Therapeutic education and follow-up”, patients are encouraged to get rid of the idea that they give to a third party full responsibility for their disease. The follow-up scheme favours patients’ empowerment and floats the idea that they can negotiate on some items. Theoretically, there would be no monopoly on medical expertise anymore, and patients suffering from chronic illness would gain some autonomy through learning, turning more responsible in the process. To a certain extent, they would become their own guides.

“Our worst fear is that patients catch an infectious disease while they’re low on white cells. So to these patients… yes, we hammer home to these patients that “antibiotics are automatic” (recommendations issued by French health insurance) [she chuckles] when they have a fever. But we try to pass on that message in a positive manner […]. We won’t say “OK, your blood cells count is 200 so if you feel feverish take antibiotics”. Hell no! Instead, we say to patients “OK, your blood cells count is 200 so if this weekend you feel kind of feverish, what do you think you should do?”. If we just deliver the info without doing some testing afterwards, we will never know if the message has hit home. At least it allows to… I mean, what I want to achieve through this follow-up scheme is that all my patients are autonomous after their second round of therapy. […] And therapeutic education somehow helps to reach that goal, I think. If I had not been trained in therapeutic education, I would not pass information on to patients the same way. Because our default mode is to do everything in their place. First this is what we… but patients have their own role to play and we must respect that if we want that they receive info properly, then remember it, repeat it and eventually use it for their own sake. So it’s important.”

(Marie coordinating nurse)

On the other hand, nurses use a more “traditional” learning (or educational) method to make sure through phone contact that homebound patients manage their therapy safely. This second method is said to be “traditional” because it does not give patients any room for negotiation. Instead, it lays the groundwork for the establishment and integration of health security rules that can even go against accepted wisdoms. In the quotation above, we are presented with the well-known pattern of learning by repeating. Health professionals working in the scheme tend to consider therapeutic education as the constant repetition of health security guidelines. Patients are therefore under nurses’ control and the relationship between both is first and foremost focused on safety. And to enforce that safety, nurses ask them to repeat and obey. Under that perspective, the follow-up scheme re-enacts a “traditional” conception of therapeutic education, directly inspired from the medicine playbook. Patients are presented with a discourse backed by expertise and legitimized by the trust they place in their doctor. Trust is indeed a prerequisite for patients to acquire medical knowledge.

“We also teach patients to read their blood test results because when we talk with them, when she [the nurse in charge of the follow-up] calls the patients, she always has their results. The lab has sent her the results of the blood test patients must take on the day before the call. So the nurse can say “OK, there is this and that…” and brief them a little on the importance of being hopeful. […] So yes, patients receive education. […] Especially on the first year of their therapy. And honestly after twelve months, they know their blood composition better than their own family doctor […]. They have become as knowledgeable as their family doctor on that.”

(Arnaud haematologist)

Follow-up calls serve as an extension of doctors’ expert speech. Nurses use them to double-down on what the doctor already said during consultations. Thus, regardless of the physical distance between nurses and patients, the follow-up scheme dwells on traditional health security control scheme and on the familiar setup of medical consultations to reinforce patients’ trust. It’s safety first, at the expense of patients’ margins of manoeuvre.

After investigation, it seems that the health security control procedures and learning methods promoted during medical consultations and in the follow-up scheme have been crafted so to be attuned to people from all walks of life, and to be cognisant of the multi-faceted relationships between doctors and patients. These patients are faced with two sets of expectations: on the one hand they are invited to negotiate with health professionals and be proactive in the management of their therapy; but on the other hand, just like in traditional medical consultations, they must hand over their freedom and comply with well-honed medical procedures.

## Conclusion

We provide a minute description of how health professionals privilege two methods to keep control over patients and teach them therapy safety procedures. On the one hand, each time patients’ health is at stake, their margin of manoeuvre is restricted [29]. They are then encouraged to be self-responsible but only within well-known boundaries where they actually relinquish their power to act and put all their trust in the doctor, all in the name of health security. On the other hand, a slightly contradictory trend delegates to patients some leeway to enforce health security, even if they remain under supervision. So patients can indeed negotiate some aspects of their therapy but under predetermined conditions and in sync with the asymmetric nature of the medical relationship, a fact with which patients themselves reckon.

This investigation invites us to take into consideration the influence of knowledge- and power-sharing in the perpetuation of epistemic inequalities. These inequalities manifest themselves in the “access, acquisition and production of knowledge or ignorance” [30]. This concept invites us to consider the joint dynamics of knowledge (of patients and health professionals) and the power (power of speech and action, asymmetries and even hierarchies established between individuals and their knowledge) of the people who hold them [31]. To the extent that the dissemination of medical guidelines on good practice by healthcare professionals transmits to patients the behaviours and skills required to ensure the safety of their therapy, it appears to be a sure way of combating epistemic inequalities. However the wide range of personalities among patients can be, or is, a breeding ground for health inequalities: depending on their profile, patients will be restricted in their access to knowledge or see their own knowledge being almost nullified; conversely, some patients will embrace the asymmetric relationship in what otherwise is a collaboration with their doctor, because at that moment of their chronic-illness trajectory [25], they need that imbalance of power.

This article shows how difficult it is to change the relationship between patients and health professionals, and the distribution of knowledge and power among them, even in a context that promotes patient participation. It appears recommended promote shared decision-making, allowing patients a voice in their treatment plans with accessible tools for informed choices. Also, our study suggests for health professionals to ensure comprehensive informed consent processes with clear explanations and adequate time for review. Finally, it is advisable to encourage education and communication adapted to patients’ profiles and on the relationship built during medical consultation to empower patients through learning. However altering the power dynamic between patients and healthcare professionals remains a challenging endeavor.

## Supporting information

S1 Checklist (DOCX)
